# VERIFICATION OF PAIN MEDICATION ADHERENCE IN OLDER ADULTS USING INTERACTIVE VOICE REMINDERS

**DOI:** 10.1093/geroni/igz038.3371

**Published:** 2019-11-08

**Authors:** Marcia Shade, Kyle Rector, Kevin Kupzyk

**Affiliations:** 1 University of Nebraska Medical Center College of Nursing, Omaha, Nebraska, United States; 2 University of Iowa, Iowa City, Iowa, United States

## Abstract

Adherence to analgesics needs to be monitored to ensure optimal pain management and avert adverse events among older adults. mHealth reminders may encourage adherence behavior, but it is unclear if medication use ensues following the reminder. The purpose of this study was to trial the use of medication event monitoring to verify the initiation of scheduled pain medication after an mHealth reminder. Methods: N=15 adults 55 and older created Google Assistant reminders to take their scheduled pain medication and write in a pain diary. A sub sample of n= 5 participants used a Medication Event Monitoring System Cap with their scheduled pain medications over 4 weeks. Data were collected on demographics, pain severity, and medication adherence. Descriptive statistics were performed. Results: Five women with ages ranging from 56-80 years, reported pain in multiple body locations. Pain severity on average was rated at 4 and at its worst 7; with pain relief ranging from 50-90%. Adherence percentages ranged from 82% to 100%. The overall latency was M = 55 min, SD = 100 min. The average latencies varied among the 5 participants; the shortest average time was 17 minutes and the longest average time was 4.5 hours. Only 15% of pain medications were taken within 5 minutes and 64% within 30 minutes of the interactive voice assistant reminder. Conclusions: It is important to ensure a behavioral intervention promotes the desired outcome. Medication event monitoring systems may help to identify non adherent behaviors when using mHealth interventions to promote pain medication adherence.

